# An Integrated Interferometric Fiber Optic Sensor Using a 638 nm Semiconductor Laser for Air-Water Surface Velocity Measurements

**DOI:** 10.3390/s23041795

**Published:** 2023-02-05

**Authors:** Ran Song, Xinyu Zhang, Lili Jiang, Zhijun Zhang, Zhigang Qiao, Xianglong Hao, Juan Su, Chenxu Lu, Guangbing Yang, Xuejun Xiong, Liyuan Gao, Chi Wu

**Affiliations:** 1 Institute of Marine Science and Technology, Shandong University, Qingdao 266237, China; 2 Southern Marine Science and Engineering Guangdong Laboratory (Guangzhou), Guangzhou 519000, China; 3 First Institute of Oceanography, Ministry of Natural Resources, Qingdao 266061, China; 4 Shandong Provincial Center for In-Situ Marine Sensors, Aixsensors, Dezhou 266101, China

**Keywords:** velocity measurement, laser interference, fiber optic sensor, sensor systems and applications

## Abstract

An integrated interferometric fiber optic velocimetry sensor has been proposed and demonstrated at the central wavelength of 638 nm. The sensor is based on the principle of two laser-beams’ interference. The light signal scattered from the particles or vapor is demodulated to measure the water surface velocity and water vapor velocity. Three velocity measurement experiments are carried out to measure the velocity, and the experimental data shows that the velocity increases linearly in the range of 4 mm·s^−1^ to 100 mm·s^−1^, with a slope of linear fitting curve of 0.99777 and the R-Square of 1.00000. The velocity calculated from frequency shift fits well with the reference velocity. The maximum average relative error in the three velocity measurements is less than 2.5%. In addition, the maximum speed of 4.398 m·s^−1^ is confirmed in the rotating disk calibration experiment, which expands the sensor’s velocity measurement range. To solve the problem that it is difficult to directly measure the velocity of small-scale water surface flow velocity, especially from the aspect of the low velocity of air-water surface, the interferometric fiber optic sensor can be applied to the measurement of water surface velocity and wind velocity on the water surface.

## 1. Introduction

Air-water surface velocity measurement has broad applications in many areas such as marine science research, oil spill prediction, search and rescue, coastal zone management, hydrology, flood forecast and ocean current dynamics study [1,2]. The marine environment, including the water body, the atmospheric space above the surface of the water, the water floor and the coastal and estuarine areas, are closely related and affected by the ocean. Studies of ocean surface flow are of great scientific significance for a deeper understanding of the interaction between ocean, land and atmosphere, and for the early warning of marine disasters. Surface velocity of the ocean is a crucial variable for ocean dynamics and surface velocity data is of pressing need for assimilation into ocean circulation models [3].

There are many water surface velocity measurement methods, including in-situ measurement and remote sensing [2,3,4,5,6,7]. These methods are promising complementary approaches for observing flow fields in small and large areas.

Fujita et al. proposed large-scale particle image velocimetry (LSPIV) for surface flow field observations in the 1990s. It has been successfully applied to various investigations of real and simple fluids in laboratories and fields [6]. A general theory for Doppler centroid measurements was proposed for ocean surface velocity measurements from space in 2005 [3]. Glomb et al. proposed a flow velocity estimation method, based on the particle image velocimetry (PIV) technique [7], but the accuracy of the calculated velocity field is limited. Underwater particle image velocimetry and acoustic Doppler velocimetry were used together for speed measurement [5]. The velocity and wave spectra of surface currents were measured via radar by detecting back-scattering of radio waves from a moving rough water surface [2,4].

Laser Doppler velocimetry (LDV) was reported for the first time by Yeh and Cummins in 1964. The classical reference optical path model has been used for the Doppler frequency shift signal measurement caused by flow [8]. The LDV system was demonstrated for non-invasive velocity measurement, with high accuracy, fast response, large dynamic range and strong ability to resist external interference [9,10]. The velocity profile of Poiseuille flow measurement was achieved using a laser Doppler velocimetry system with a focus tunable lens [11].

A Doppler anemometer employing semiconductor lasers demonstrated variable measurement distance for wind speed [12]. Laser Doppler velocimetry can be miniaturized and compacted by using semiconductor lasers and optic fibers [10,13].

However, it has been a challenge for some time to directly measure small-scale water surface flow velocity, especially for air-water surface velocity. Traditional laser Doppler has limitations in low speed measurement. Two-beam interferometric velocimetry, based on the principle of differential laser Doppler measurement, has superior advantages for low speed measurement. This manuscript proposes and experimentally demonstrates an integrated interferometric optic velocity sensor employing a semiconductor laser. The optic back-scattering signal from particles in the water or air was detected to demodulate the velocity of water or air. Since the distance between interferometric fringes and sensor is controllable, the sensor has the advantages of being non-invasive, high precision, and with high spatial and temporal resolution. The device is, thus, suitable for small-scale flow measurement of air-water surface velocity.

## 2. Principle and Measurement System

Two separated beams from one laser are focused after passing through the lens and the interferometric fringes are generated. When particles in the water or air pass through the interferometric fringes at the light focus point, the light is scattered and received by the photodiode. By processing the electric signal collected from the photodiode, the velocity of water or air is obtained [14,15]. 

**Figure 1:**
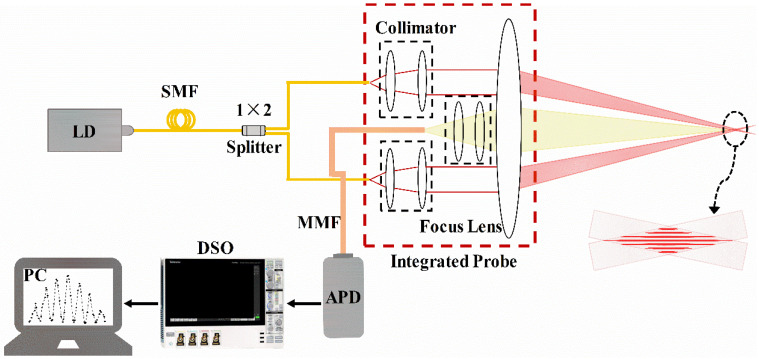
Diagram of velocity sensor.

**Figure 2:**
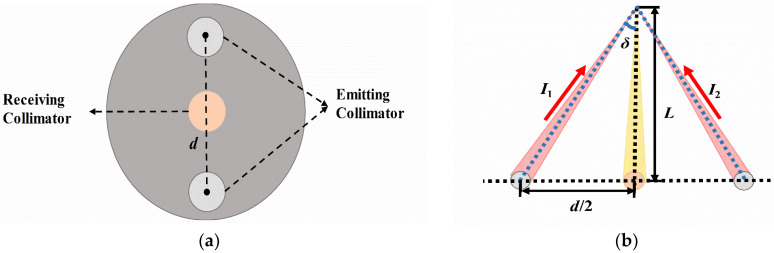
Schematic diagram of: (**a**) Integrated optic probe; (**b**) Light paths in the measurement space.

A semiconductor laser diode (LD, Integrated Optics, 0638L-23A-NI-AT-NF) with an output power of 60 mW and a central wavelength of 638 nm was used. Its light beam was coupled into a pigtail (single-mode fiber, SMF) and then butt-coupled into the beam splitter to generate two beams with equal intensity. The two beams of light became collimated beams after passing through the collimators (emitting collimator). Then they were focused by a plano-convex lens (with a focal length (F) of 250 mm and diameter of 101 mm). The two interference beams were focused at the measurement point and the interferometric fringes were generated in the measurement volume. The distance (*d*) between the two collimating beams is set to be 76 mm. The measurement distance (*L*) is from the focus lens (FL, plano-convex lens) to the velocity measurement point. Only when the particles pass through the interferometric fringes is light scattered back to the plano-convex lens, becoming parallel light, and collected in the multimode fiber (MMF) via a collimator (receiving collimator). The scattered light was transmitted into an avalanche photodetector (APD, Thorlabs, APD430A2/M) and converted into an electrical signal. Then the electric signal was collected by a digital storage oscilloscope (DSO) with a sampling rate of 12.5 MS/s and inputted into a personal computer (PC) for data processing. The diagram of the whole system is shown in Figure 1. Both emitting collimators with SMF pigtails and receiving collimator with MMF pigtail are integrated on a mechanical disk, as shown in Figure 2a.

The light intensity distribution of the interference fringes at the measurement distance *L* is described by the following Equation (1) at the interfering volume [16].

(1) $$I=I_{1}+I_{2}+2\sqrt{I_{1}I_{2}}\cos\Delta \varphi$$

where *I*_1_ and *I*_2_ are the light intensities emitted from the plano-convex lens, as shown in Figure 2b, and △*φ* is the phase difference between the two beams.

The whole interference system presents a symmetrical structure. Since the light from the laser diode is split by the beam splitter, two light beams have the same frequency and constant initial phase difference. For this case, *I*_0_ = *I*_1_ = *I*_2_, we can get [16]

(2) $$I=2I_{0}(1+\cos\Delta \varphi )\;=4I_{0}\cos^{2}\frac{\Delta \varphi }{2}$$

When the phase difference is satisfied with

(3) Δφ=2mπ, (m=0,±1,±2,⋯)

The maximum light intensity appears, where *I* = 4*I*_0_ [16].

When the phase difference is satisfied with

(4) Δφ=(2m+1)π, (m=0,±1,±2,⋯)

The minimum light intensity appears, where *I* = 0 [16].

There is an angle between the two emitted beams, and the phase difference or optical path difference between the two beams arriving at each point of the interference field will lead to different light intensities. The interference fringes pattern of light and dark will appear.

When the particles pass through the alternating light and dark interference fringes, the intensity of the scattered light changes over a period and a signal with a frequency is generated. When velocity of the particles changes, period of the scattered light changes. The light intensity of a signal is related to both frequency and amplitude. This characteristic is embodied, in that the frequency change of the moving particle passing through the fringes corresponds to the frequency change of the light intensity signal, and the intensity signal amplitude of the scattered light corresponds to the Gaussian distribution of the light intensity in the measurement volume. Based on these properties, an expression for the signal strength received by the photodetector could be derived as [17]:

(5) $$\begin{array}{l} i(t)=i_{d}\exp\left\{-{\left[\frac{2\sqrt{2}(t-t_{0})}{\tau }\right]}^{2}\right\}\\ +i_{a}\exp\left\{-{\left[\frac{2\sqrt{2}(t-t_{0})}{\tau }\right]}^{2}\right\}\cos\left[2\pi f(t-t_{0})\right] \end{array}$$

The signal is composed of two parts: one is the base with lower frequency, whose amplitude is *i_d_*; the other is the cosine function, whose amplitude is *i_a_*. This cosine part of the signal is overlapped on the base.

(6) $$\tau =\frac{N_{f}}{f}$$

*τ* is the time for the moving particles passing through the interference fringes. *N_f_* is the number of fringes. *f* is the frequency shift.

(7) $$t=\frac{1}{f_{s}}$$

*f_s_* is the sampling frequency.

(8) $$t_{0}=\frac{a}{f_{s}},\;a=a_{1},\;a_{2},\;\ldots ,\;a_{20},\;\ldots ,\;a_{1}=0$$

*t*_0_ is the arrival time of the particle.

**Figure 3:**
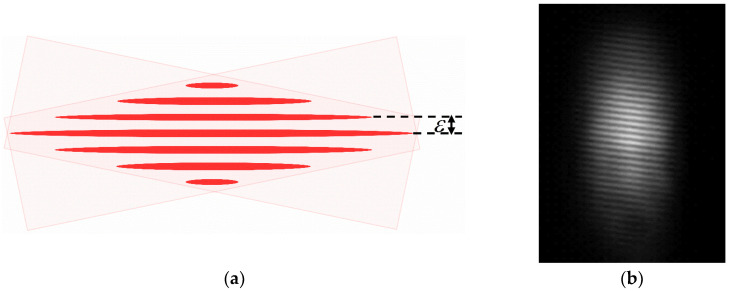
The fringe pattern. (**a**) Schematic of fringes; (**b**) The fringe pattern taken by a CMOS camera.

The intensity distribution of interference fringes is also measured in our experiments. The fringe pattern, shown in Figure 3b, is taken by a compact scientific digital camera (Thorlabs, CS505MU) in the experiment. The camera is a complementary metal oxide semiconductor (CMOS) monochrome placed at the focal point of the two beams to record light interference fringes. 

The velocity demodulation is carried out based on the fringe pattern model. From Figure 1 and Figure 2b, the fringe spacing *ε* could be obtained [18],

(9) $$\varepsilon =\frac{\lambda }{2\sin\delta }\approx \frac{L\lambda }{d}$$

And the velocity is expressed as [18]:

(10) v=εf

where *ε* is the fringe spacing between the centers of two bright fringes, *λ* is the wavelength of the light, *δ* is the half-angle between the two interference beams and *f* is the frequency shift that can be obtained from the photodiode by the electric signal. 

The larger the *L*, the less the influence of the velocimetry on the flow field. However, water has a strong absorption of light. If *L* is too large, the scattered light to the detector will be too weak to be detected. A velocimetry with a smaller size would allow a larger *L* for unperturbed measurement. The interference fringe spacing *ε* is inversely proportional to *d* according to Equation (9). When *d* is smaller, *ε* is larger and the interference fringes are easier to be distinguished. The requirement for the response time of the detector is lower. A larger speed range can be measured. However, *d* is limited by the size of three collimator lens mounts, as shown in Figure 2a. Considering both practical engineering design and optimized performance, *d* is set to be 76 mm. 

To calculate the velocity using Equation (10), *f* is obtained using the reciprocal of the time interval Δ*t*, which is the time of the particle passing through the two adjacent bright fringes. This could be expressed as:

(11) $$f=\frac{1}{\Delta t}$$

## 3. Results and Discussion

**Figure 4:**
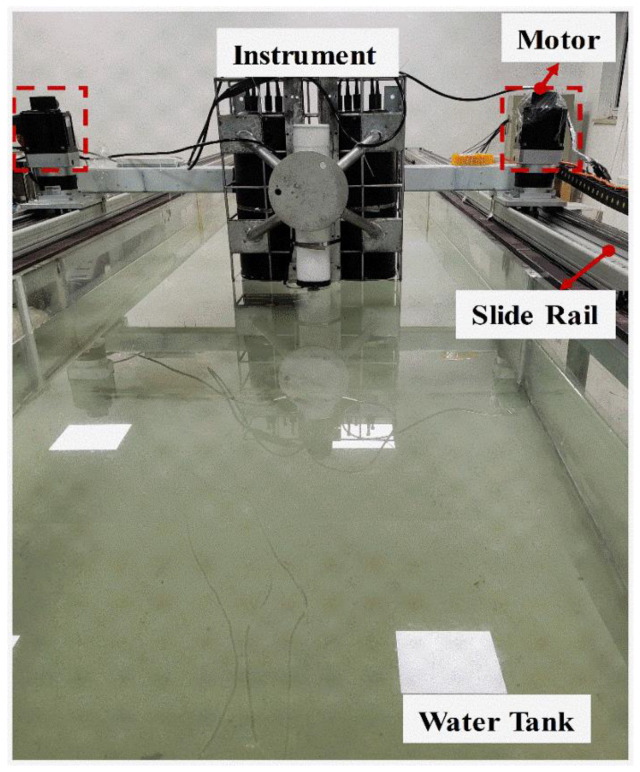
Motorized slide rail on the water tank for velocity calibration and measurement.

To calibrate the velocity sensor, a high precision motorized slide rail on the water tank (1.2 m × 1 m × 8 m) was used as the reference velocity. The sensor was attached to the motorized slide rail and moves along with the slide rail to generate a reference linear speed with high precision, as shown in Figure 4.

Monodispersed titanium dioxide spheres (with a diameter of 50 μm, refractive index of 1.56 and density of 1.03–1.05) were placed in the water as particles to form a dilute colloidal suspension (1 part solid to 1 × 10^6^ parts H_2_O by volume).

**Figure 5:**
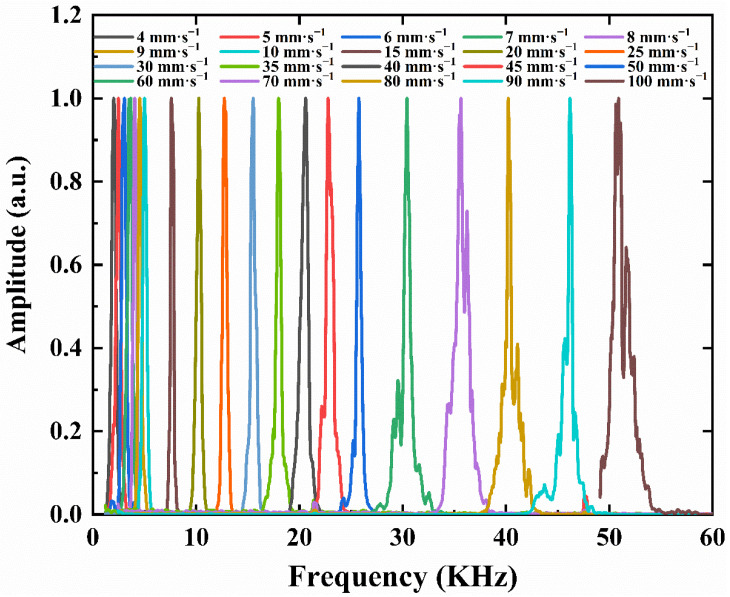
Frequency shift wave packet of the backscattering light when the velocity of the slide rail changes from 4 mm·s^−1^ to 100 mm·s^−1^.

The frequency shift of the backscattering light is shown in Figure 5 when the velocity of the slide rail changes from 4 mm·s^−1^ to 100 mm·s^−1^. The measured relative velocity between the velocity sensor and water is calculated from frequency shift according to Equation (10).

### 3.1. Experimental Results of Rotating Disk Calibration

In order to reduce velocity calculation errors in measuring *L*, *d*, and *ε*, it is necessary to calibrate the velocity sensor using a line speed generated by a rotating disk. The line speed of the rotating disk is written as 2*πrF_d_*, where *r* is the distance from the light spot to the center of the rotating disk and *F_d_* is the rotation frequency of the rotating disk.

**Figure 6:**
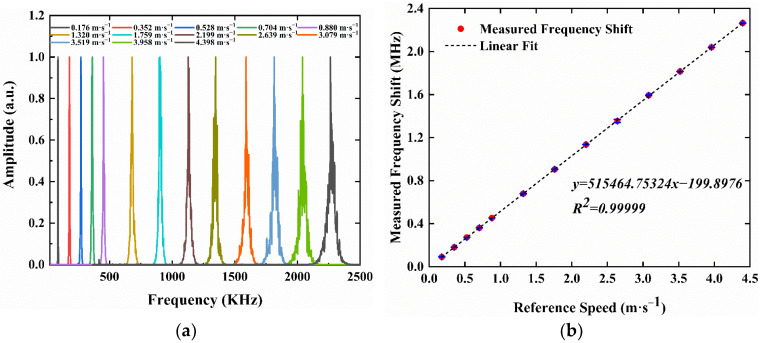
Measurement and fitting results of the speed of the rotating disk. (**a**) Frequency shift wave packet of the measured scattering light with a velocity range from 0.176 m·s^−1^ to 4.398 m·s^−1^; (**b**) The relationship between measured frequency shifts and reference speeds.

The scattering light signals were collected by APD and further processed by a fast Fourier transform (FTT) algorithm. The frequency shift wave packets of the scattering light at different line speeds of rotating disk are shown in Figure 6a. By rearranging the results of Figure 6a, the relationship between the measured frequency shift and the reference speed of the rotating disk is shown in Figure 6b. For speed range from 0.176 m·s^−1^ to 4.398 m·s^−1^, a good linear fitting result of R^2^ = 0.99999 is obtained. The blue error bar in Figure 6b is the result from three repeated measurements.

According to Equation (12), the inverse of the slope *k* of the fitted curve given in Figure 6b is corresponding to the fringe spacing *ε*. When *k* = 515,464.75324, the fringe spacing of 1.9399968547 μm is obtained.

(12) $$\varepsilon =\frac{1}{k}$$

### 3.2. The Speed Measurement Results from the Motorized Slide Rail on the Water Tank

**Figure 7:**
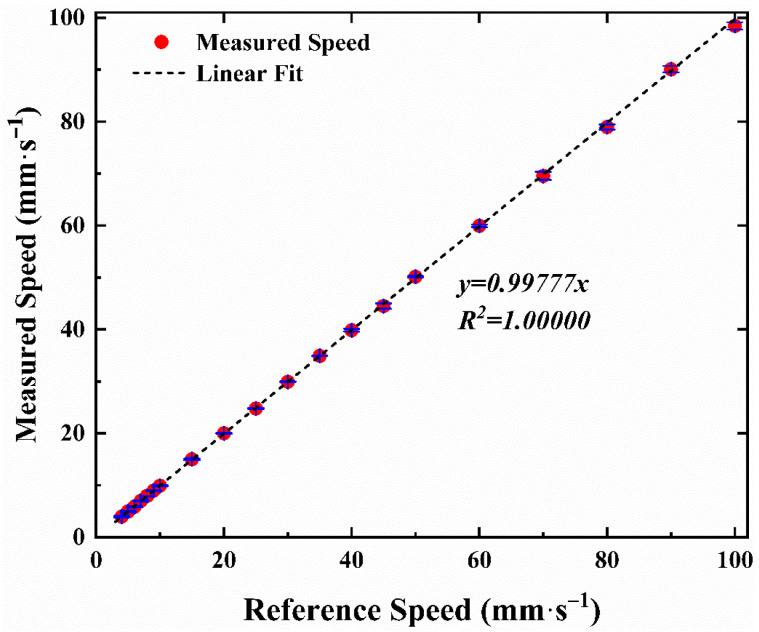
The measured velocity against the reference velocity of the slide rail in the range of 4 mm·s^−1^ to 100 mm·s^−1^ (R^2^ = 1.00000).

The speed of a high-precision slide rail driven by an electric motor on the water tank was used as the reference speed for the measurement of air-water surface velocity. The velocity sensor was placed on the slide rail and the interference fringes were focused on the surface of the water. As the slide rail moved at different speeds, the light scattering signal was collected by the receiver system and processed by noise filtering and FFT to obtain frequency shift. The frequency signal was then calculated by Equation (10) to obtain speed information. The measured velocities against the reference velocities of the slide rail are compared in Figure 7, in the range of 4 mm·s^−1^ to 100 mm·s^−1^. The minimum measured speed is limited by the electric motor of the slide rail. The blue error bar is also the result of three repeated measurements. The linear fitting curve gives a slope of 0.99777, with the R^2^ of 1.00000.

The relative error of the three repeated measurements is given by,

(13) $$R=\frac{x-\bar{x}}{\bar{x}}\times 100\%$$

where R is the relative error, *x* is the measured data obtained in the experiment and $\bar{x}$ is the reference velocity.

**Figure 8:**
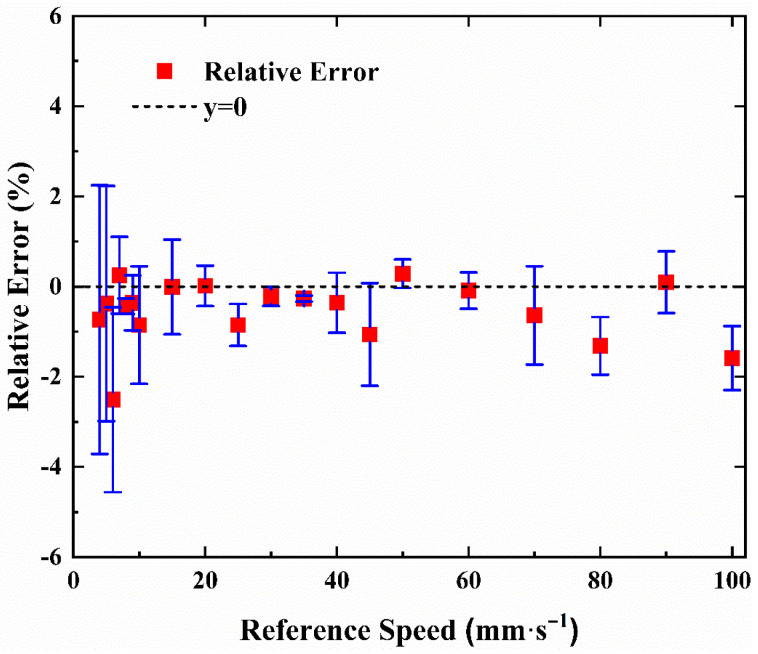
Relative error of the three repeated measurements.

The average relative error of the three repeated measurements is shown in Figure 8. The absolute value of the maximum average relative error of three repeated measurement is less than 2.5%. 

Equations (14) and (15) give the calculated population variance and standard deviation of relative error, which are 4.4370 × 10^−5^ and 6.6611 × 10^−3^, respectively. These results show that our measurements are relatively stable.

(14) $$s^{2}=\frac{1}{n}\sum_{i=1}^{n}(n_{i}-\bar{n}{)}^{2}$$

(15) $$\sigma =\sqrt{\frac{1}{n}\sum_{i=1}^{n}(n_{i}-\bar{n}{)}^{2}}$$

where s^2^ is the population variance, *σ* is the standard deviation, *n* is the number of samples, *n_i_* is any sample, which is the average of relative errors of three times of measurement, and $\bar{n}$ is the average of the samples.

**Figure 9:**
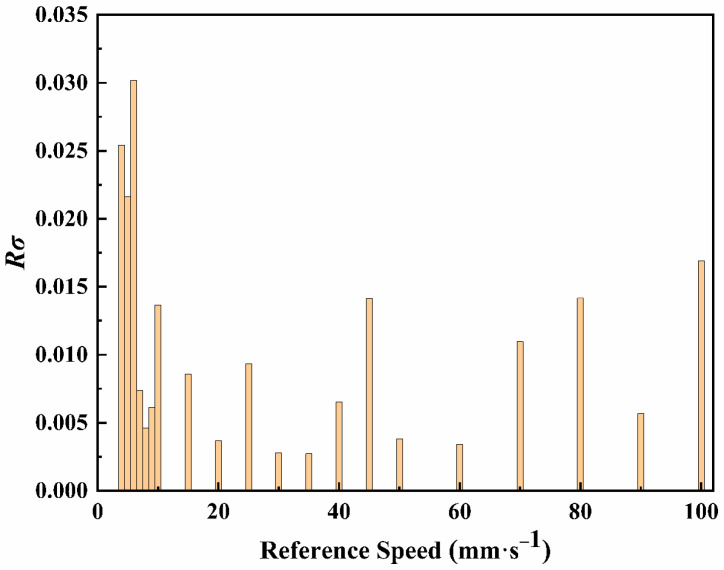
The relative standard deviation of three repeated measurements at each velocity.

The relative standard deviation *Rσ* of the three repeated measurements at each velocity value are calculated in Figure 9 using Equation (16).

(16) $$R\sigma =\frac{\sqrt{\frac{{\left(x_{1}-\bar{x}\right)}^{2}+{\left(x_{2}-\bar{x}\right)}^{2}+{\left(x_{3}-\bar{x}\right)}^{2}}{3}}}{\bar{x}}$$

where *x*_1_, *x*_2_, and *x*_3_ are the velocity values of three times of measurement corresponding to each velocity, respectively, and $\bar{x}$ is the reference velocity.

### 3.3. The Speed Measurement of Vapor

**Figure 10:**
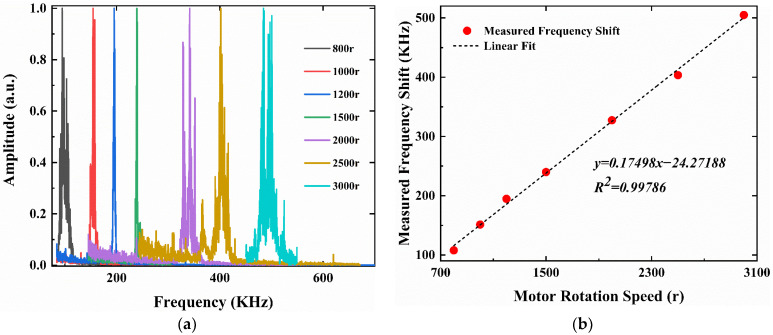
Measurement and fitting results of the speed of vapor with motor rotation speed range of 800 r to 3000 r. (**a**) Frequency shift wave packet; (**b**) The relationship between measured frequency shifts and motor rotation speeds.

In addition to water surface velocity measurements, the velocity of the gas with a large water vapor content was also measured to demonstrate that the air velocity above the water surface could also be measured by this sensor. A wind tunnel (Kanomax, X5605) was deployed to generate air flow speed. By varying motor rotation speed (r), the suction force was adjusted to make the water vapor leave the humidifier at different speeds. The light beams were focused on the water vapor to measure its speed. The scattered light was collected by the APD and further processed to obtain the frequency shifts. The frequency shift wave packets corresponding to different rotation speeds are shown in Figure 10, demonstrating a good correlation and signal-noise ratio. Since the wind tunnel was not calibrated, a relative speed measurement with a good linear relationship is obtained instead of the absolute speed.

Table 1 presents the performance comparison of various velocity measurement techniques, including Large-scale particle image velocimetry (LSPIV), laser Doppler velocimetry (LDV), and dense optical flow method. In the table, the maximum velocity refers to the maximum measured velocity in the experiment given in the published paper. Compared with the velocimeters mentioned above, our proposed interferometric fiber optic sensor using a 638 nm semiconductor laser has the advantages of high resolution, large dynamic range and compact size, and is more suitable for small-scale air-water surface flow velocity measurement.

## 4. Conclusions

This paper proposed and experimentally demonstrated a fiber optic interferometric velocimetry using a compact semiconductor laser at a central wavelength of 638 nm to measure the air-water surface velocity and air vapor velocity. The air-water surface velocity was measured in the range of 4 mm·s^−1^ to 100 mm·s^−1^, with a good linear fitting slope of 0.99777 and R^2^ of 1.00000, and an average relative error of less than 2.5%. The maximum speed of 4.398 m·s^−1^ generated by a rotating disk was achieved.

This sensor could have potential application for in-situ air-water surface flow speed measurement, with the properties of non-invasiveness, rapid dynamic response time and compactness.

## Figures and Tables

**Table 1:** Performance Comparison of Various Velocimeters.

Working Principle	Resolution	Maximum Velocity	Ratio between the Maximum Velocity and Resolution	Relative Error	Small Scale	Cross-Medium
LSPIV [6]	20 mm·s^−1^	4 m·s^−1^	2 × 10^4^%	5%	No	No
LDV (argon ion laser) [9]	100 mm·s^−1^	4.45 m·s^−1^	4.45 × 10^3^%	\	Yes	Yes
Fiber optic confocal LDV (*λ* = 1550 nm) [10]	7.5 mm·s^−1^	0.35 m·s^−1^	4.667 × 10^3^%	\	Yes	No
LDV (*λ* = 660 nm) [11]	1 mm·s^−1^	2.4 m·s^−1^	2.4 × 10^5^%	3.5%	Yes	Yes
Dense optical flow [19]	200 mm·s^−1^	0.5 m·s^−1^	2.5 × 10^2^%	6.5%	Yes	No
This paper	1 mm·s^−1^	4.398 m·s^−1^	4.398 × 10^5^%	2.5%	Yes	Yes

## Data Availability

Not applicable.
